# Importance of Radioactive Labelling to Elucidate Inositol Polyphosphate Signalling

**DOI:** 10.1007/s41061-016-0099-y

**Published:** 2017-01-18

**Authors:** Miranda S. C. Wilson, Adolfo Saiardi

**Affiliations:** Medical Research Council Laboratory for Molecular Cell Biology, University College London, Gower Street, London, WC1E 6BT UK

**Keywords:** Radioactivity, Inositol, Pyrophosphates, Metabolism, Phosphate

## Abstract

Inositol polyphosphates, in their water-soluble or lipid-bound forms, represent a large and multifaceted family of signalling molecules. Some inositol polyphosphates are well recognised as defining important signal transduction pathways, as in the case of the calcium release factor Ins(1,4,5)P_3_, generated by receptor activation-induced hydrolysis of the lipid PtdIns(4,5)P_2_ by phospholipase C. The birth of inositol polyphosphate research would not have occurred without the use of radioactive phosphate tracers that enabled the discovery of the “PI response”. Radioactive labels, mainly of phosphorus but also carbon and hydrogen (tritium), have been instrumental in the development of this research field and the establishment of the inositol polyphosphates as one of the most important networks of regulatory molecules present in eukaryotic cells. Advancements in microscopy and mass spectrometry and the development of colorimetric assays have facilitated inositol polyphosphate research, but have not eliminated the need for radioactive experimental approaches. In fact, such experiments have become easier with the cloning of the inositol polyphosphate kinases, enabling the systematic labelling of specific positions of the inositol ring with radioactive phosphate. This approach has been valuable for elucidating their metabolic pathways and identifying specific and novel functions for inositol polyphosphates. For example, the synthesis of radiolabelled inositol pyrophosphates has allowed the discovery of a new protein post-translational modification. Therefore, radioactive tracers have played and will continue to play an important role in dissecting the many complex aspects of inositol polyphosphate physiology. In this review we aim to highlight the historical importance of radioactivity in inositol polyphosphate research, as well as its modern usage.

## Introduction

**Fig. 1:**
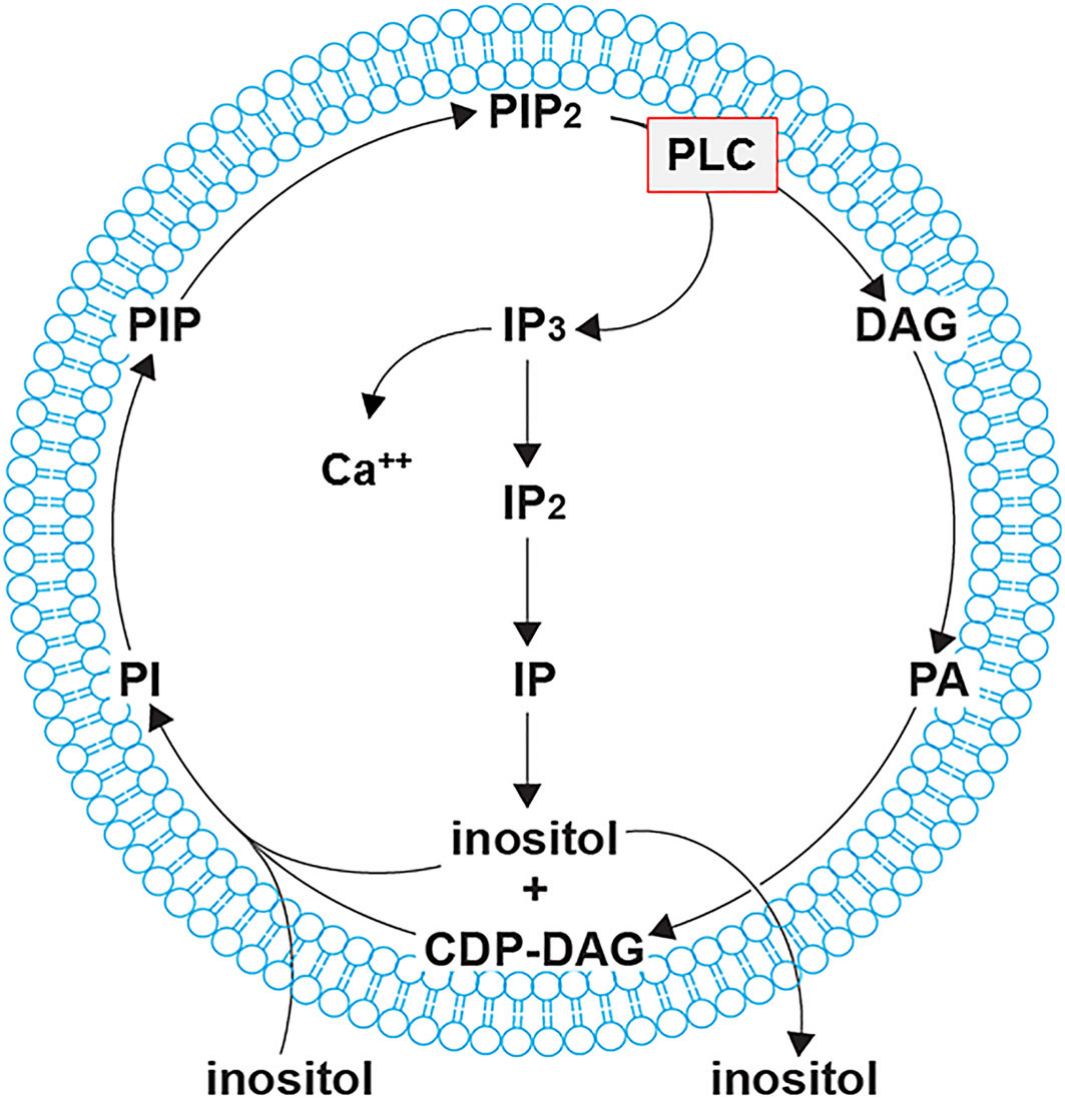
Schematic representation of the inositol cycle. Inositol acquired from the extracellular space is incorporated into lipids by the action of the phosphatidylinositol synthase (PI-synthase). The conversion of PtdIns (PI), first to PtdIns(4)P (PIP) and then to PtdIns(4,5)P_2_ (PIP_2_), generates the substrate for phospholipase C (PLC, *boxed* in *red*). Once receptor activation occurs, PLC generates two second messengers: the (plasma) membrane-resident diacylglycerol (DAG) and the calcium (Ca^2+^) release factor Ins(1,4,5)P_3_ (IP_3_). The latter is converted back to inositol via two dephosphorisation steps, closing the cycle. The inositol cycle is particularly active in stimulated mammalian cells. The Hokin “PI response” [8, 9] measures the [^32^P] taken up by the cell and its conversion to [^32^P] γATP, with the subsequent radioactive phosphorylation of DAG to phosphatidic acid (PA). PA is then reattached to inositol, creating radioactive PI. For graphical reasons, inositol is abbreviated here as “I” instead of “Ins”, and phosphatidylinositol as “PI” instead of “PtdIns”

Inositol polyphosphates comprise a vast and multifaceted family of cellular metabolites. The size of the family is explained by the ability to combinatorially substitute the six hydroxyls of the *myo*-inositol ring with phosphate moieties: mathematically, 64 such combinations are possible [1]. This number is in fact an underestimate, as diphosphate (or pyrophosphate) moieties also exist [2– 4]. Among these myriad inositol polyphosphates, without doubt the most famous is the calcium release factor Ins(1,4,5)P_3_, the prototypical second messenger. Hydrolysis of the lipid bond of PtdIns(4,5)P_2_ by phospholipase C (PLC) following receptor activation to release the water-soluble Ins(1,4,5)P_3_ and the lipid diacylglycerol (DAG) is a textbook example of signal transduction (Fig. 1) [5, 6]. It results in Ins(1,4,5)P_3_ binding to the InsP_3_ receptor and the consequent release of calcium from intracellular stores [7].

This PLC activity was indirectly assayed during the first discovery of receptor-stimulated inositol polyphosphate metabolism. In the early 1950s, the Hokin husband and wife team were studying RNA metabolism using radioactive orthophosphate [^32^P] metabolic labelling, when they discovered an increase in cellular incorporation of radioactivity when pancreatic slices were stimulated with acetylcholine. Surprisingly, however, the large majority of radioactivity was not incorporated into nucleic acids but into the inositol-containing lipids called phosphoinositides (PI or PtdInsP, phosphatidylinositols); this acetylcholine-stimulated [^32^P] incorporation was therefore termed the “PI response” [8, 9]. It was not until three decades later that the PI response was determined to be part of PLC activation [6, 10] (Fig. 1). The [^32^P] taken up is initially incorporated into ATP and other nucleotides. Radioactive phosphorylation of DAG to phosphatidic acid (PA) allows its reattachment to inositol, creating PtdIns that is further phosphorylated, generating the radioactive PtdInsP/“PI” that the Hokins observed, and completing what is now known as the inositol cycle (Fig. 1).

This short historical background highlights the fundamental importance of radioactive phosphate labelling in the birth of the phosphoinositide and inositol polyphosphate signalling research fields. Equally, everyone is aware of the historical—and indeed, current—importance of radioactive labelling in nucleic acid research [11]. Without radioactivity-based methods, molecular biology would not have emerged and we would not be in the post-genomic era of biomedical research. It is not an overstatement to say that without radioactivity, the advances in biomedical science and consequent improvements in human health of the past century would not have been achieved. The ongoing importance of radioactive labelling to inositol polyphosphate research should also not be underestimated.

The general perception of “radioactivity” is one of fear, and understandably so, considering famous disasters such as those at Chernobyl or Fukushima [12]. However, using very low trace levels of radioactivity in a highly controlled research environment is safe: many protection and monitoring measures are available [13, 14]. Young researchers should therefore embrace radioactivity-based techniques, as they offer unique research opportunities, even in this twenty-first century, and especially in the inositol polyphosphate research field. Inositol polyphosphates, being unable to absorb UV/visible wavelength light, cannot be detected by absorbance or fluorescence methods. Thus the study of their metabolism and physiological functions has only been possible through radioactivity experiments. The current essay will focus primarily on the importance of radioactive phosphate labelling ([^32^P] and [^33^P]) in inositol polyphosphate biology. We will discuss the use of radioactive orthophosphate tracers to study the cellular metabolism of inositol polyphosphates, as well as the biochemical synthesis and purification of inositol polyphosphates radiolabelled at specific positions on the inositol ring. To provide a complete view of relevant radioactive methods, we will also concisely describe the use of radioactive hydrogen (tritium [^3^H]) and carbon (carbon-14 or [^14^C]) labelling techniques.

##  Phosphoinositides and Inositol Phosphates

**Fig. 2:**
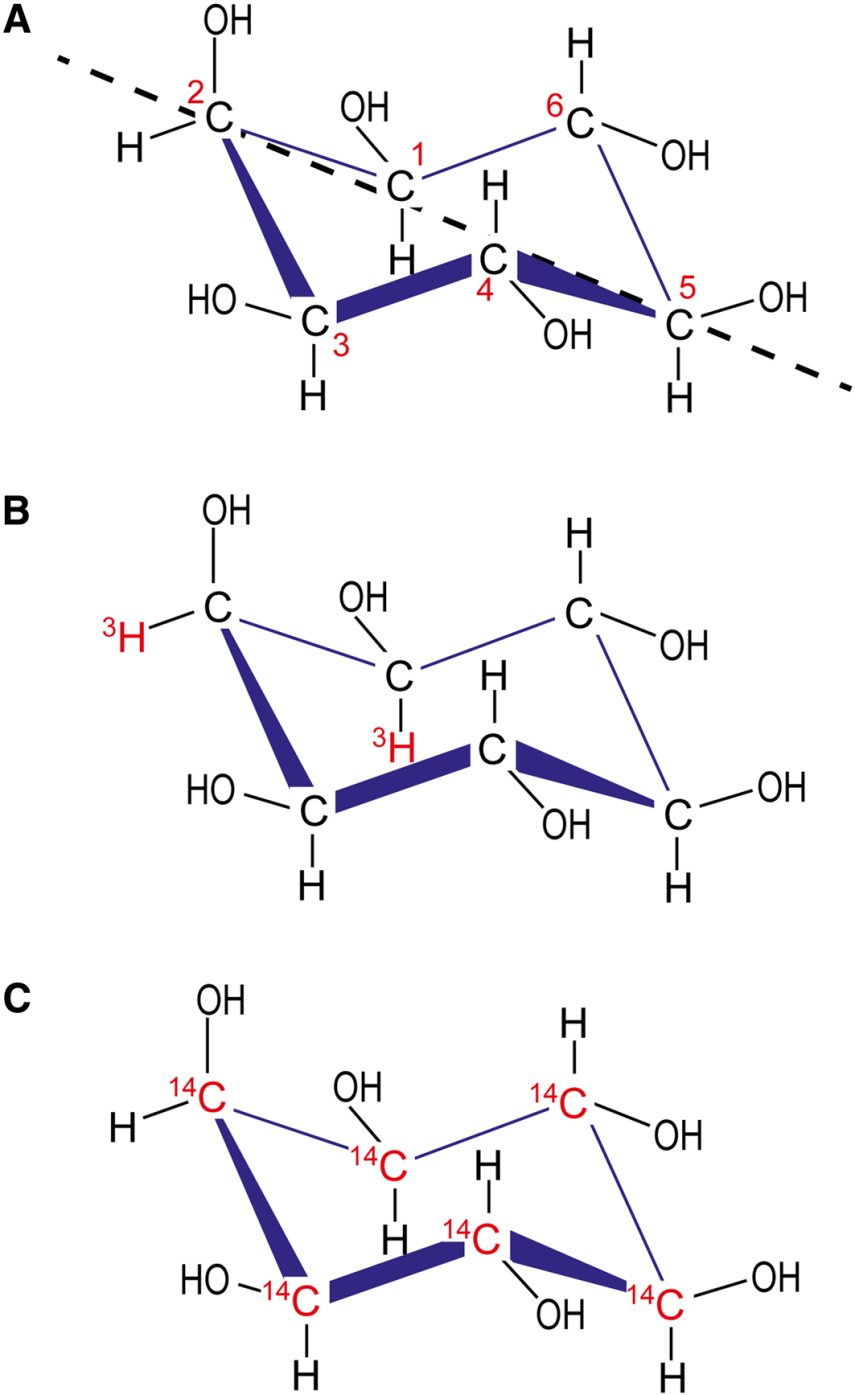
*myo*-Inositol structure and its radiolabelled derivatives. While nine stereoisomeric configurations of inositol are possible, the structure of *myo*-inositol is depicted in (**a**), referred to in the review simply as inositol, since it is by far the most common and biologically relevant form of inositol. The modern D-numbering system for inositols is counterclockwise as viewed from above and assigns the single axial hydroxyl group of *myo*-inositol to the carbon in position 2, while the other five hydroxyls are equatorial. *myo*-Inositol possesses an axis of symmetry through carbons 2 and 5 (*dashed line*), making positions 1,3 and 4,6 enantiomeric. The most common commercially available tritium-labelled inositol (**b**) possess the [^3^H] radiolabelled in position 1 and/or 2, while in [^14^C]inositol the radiolabel is uniformly distributed (**c**)

Before discussing radioactive labelling methods in inositol polyphosphate research, we must briefly introduce these molecules and the metabolic pathways connecting them. As it is not the main scope of this essay, discussion of inositol polyphosphate metabolism will be highly simplified; the interested person is encouraged to read the following more comprehensive reviews [15–18]. The carbon backbone of *myo*-inositol (hereafter simply called “inositol”) is by far the most common and biologically relevant of the naturally occurring stereoisomers. It is also the structural building block for the inositol polyphosphates. In its favoured chair conformation, inositol has five equatorial and one axial hydroxyl group (Fig. 2a) [19]. This axial hydroxyl is found at the carbon in position 2, using the D-numbering convention for cyclitols.

**Fig. 3:**
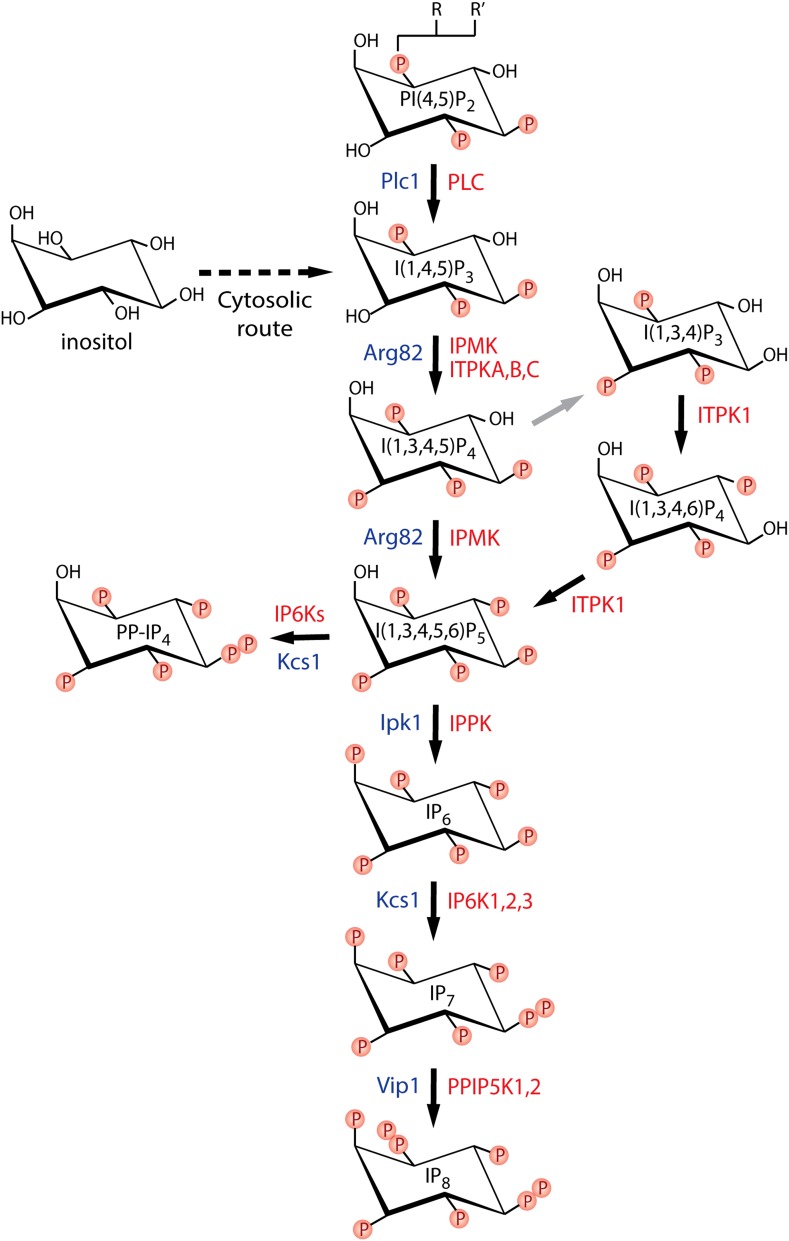
Inositol polyphosphates synthetic pathway. The synthesis of higher phosphorylated inositol polyphosphates begins with the synthesis of Ins(1,4,5)P_3_. *Saccharomyces cerevisiae* uses only phospholipase C (PLC) hydrolysis of the lipid PI(4,5)P_2_ to synthesise Ins(1,4,5)P_3_ [28], whereas *Dictyostelium discoideum* utilises the cytosolic route, of which the enzymology is not fully elucidated (*dashed line*) [110]. Ins(1,4,5)P_3_ is metabolised by ITPKA, B, or C to synthesise Ins(1,3,4,5)P_3_, which is acted on by the 5-phosphatase (*grey line*) to generate the Ins(1,3,4)P_3_ converted by ITPK1 to Ins(1,3,4,5,6)P_5_. However, this isomer of InsP_5_ can also be directly generated by IPMK from Ins(1,4,5)P_3_. InsP_5_ is converted to InsP_6_ by the IP_5_-2Kinase IPPK. Phosphorylation of InsP_6_ by the IP6Ks generates the inositol pyrophosphate InsP_7_, specifically the depicted isomer 5PP-InsP_5_, which is further acted on by PPIP5K1,2 to IP_8_, specifically to 1,5(PP)_2_-IP_4_. The IP6K enzymes can also use Ins(1,3,4,5,6)P_5_ as a substrate, generating the inositol pyrophosphate PP-IP_4_. In this figure, for visual reasons, inositol is abbreviated as “I” instead of “Ins”, and phosphatidylinositol as “PI” instead “PtdIns”. Kinases catalysing each step are indicated in *red* (human) and *blue* (*S. cerevisiae*)

The simplest function of inositol is as an osmolyte, whose cellular concentration is regulated in response to hyperosmolarity. However, more interesting functions are achieved through phosphorylation to create inositol polyphosphates. These water-soluble molecules have a complicated biosynthetic pathway in yeast and, presumably, in mammalian cells: not just sequential phosphorylation or dephosphorylation, but synthesis that is intimately linked to the metabolism of the related PtdIns lipids (Figs. 1, 3) [20, 21]. Cells can synthesise inositol de novo from glucose-6-phosphate or acquire it from the extracellular environment, allowing it to enter the inositol cycle (Fig. 1). Inositol lipid synthesis starts with the activation of PA with CTP, becoming CDP-DAG that is subsequently attached to the 1-hydroxyl of the inositol ring, forming PtdIns. This can be phosphorylated to PtdIns(4)P and then to PtdIns(4,5)P_2_, the substrate for phospholipase C (PLC), in the calcium release signalling paradigm described above. It is important to remember that the universal production of Ins(1,4,5)P_3_ by PLC does not necessarily translate into calcium signalling, as many eukaryote clades including yeast and plants do not possess InsP_3_ receptors [22]. The Ins(1,4,5)P_3_ generated by PLC activity can be dephosphorylated back to inositol and reused for PtdIns synthesis. Conversely, Ins(1,4,5)P_3_ can act as precursor for a large, diverse family of higher phosphorylated inositol polyphosphates (Fig. 3). For example, Ins(1,4,5)P_3_ is a substrate of the inositol polyphosphate multikinase (IPMK; yeast Arg82) [23–26], which is able to phosphorylate both positions 3 and 6, creating Ins(1,3,4,5,6)P_5_. This is acted on by inositol pentakisphosphate 2-kinase (IP_5_-2K or IPPK; Ipk1 in yeast) [27, 28] to create the fully phosphorylated inositol hexakisphosphate (InsP_6_ or phytic acid). Another metabolic route can also lead to Ins(1,3,4,5,6)P_5_ synthesis. Phosphorylation of Ins(1,4,5)P_3_ by ITPKA,B,C, the IP_3_-3Ks, generates Ins(1,3,4,5)P_4_ [29, 30] that is dephosphorylated by 5-phosphatases such as SHIP1 into a different InsP_3_ isomer, Ins(1,3,4)P_3_. This isomer is a substrate for ITPK1, another multikinase, which adds a phosphate group at positions 5 and 6, again resulting in an InsP_5_ species with the remaining hydroxyl group at the 2 position [31–33] (Fig. 3). Furthermore ITPK1 can also phosphorylate position 1 of the inositol ring [34, 35].

Together, Ins(1,3,4,5,6)P_5_ and InsP_6_ are the major forms of inositol polyphosphates present in mammalian cells. Despite their association with numerous cellular functions, and unlike Ins(1,4,5)P_3_, no dramatic or rapid changes in the cellular amount of either molecule are seen on receptor activation, although their relative level is modulated by neurotrophin signals [36]. However, they are not metabolically inert: Ins(1,3,4,5,6)P_5_ and InsP_6_ are substrates for the synthesis of a subfamily of inositol polyphosphates called inositol pyrophosphates [37–39]. These contain one or more high-energy phosphoanhydride (pyro) bonds as well as the phosphoesters. Two classes of enzyme synthesise inositol pyrophosphates in mammalian cells. The IP6Ks (InsP_6_ kinase) are able to pyrophosphorylate position 5 of the inositol ring, generating 5PP-InsP_5_ (diphosphoinositol pentakisphosphate, InsP_7_) from InsP_6_ or 5PP-InsP_4_ from Ins(1,3,4,5,6)P_5_ [25, 38–43]. Alternatively, PPIP5K (VIP1 in yeast) enzymes can pyrophosphorylate position 1, generating the 1PP-InsP_5_ isomer of InsP_7_ from InsP_6_ in vitro [44–47], and InsP_8_ ([PP]_2_-InsP_4_; bis-diphosphoinositol-tetrakisphosphate) from 5PP-InsP_5_ in vivo [48, 49].

The characterised inositol phosphate kinases (Table 1) offer the opportunity to label the inositol ring with radioactive phosphate simply by performing in vitro enzymatic reactions using recombinant enzyme with radioactive [^32^P]γATP. We will first introduce the radioactive isotopes available, before describing their use.

**Table 1:** Inositol phosphate kinases identified in the human and yeast (*S. cerevisiae*) genomes

Inositol polyphosphate kinase	Human	Yeast	Main enzymatic activities	References
Inositol-trisphosphate 3-kinase	ITPKA,B,C	–	Ins(1,4,5)P_3_ => Ins(1,3,4,5)P_4_	[74, 105–107]
Inositol polyphosphate multikinase	IPMK	Arg82	Ins(1,4,5)P_3_ => Ins(1,3,4,5)P_4_ Ins(1,3,4,5)P_4_ => Ins(1,3,4,5,6)P_5_ PtdIns(4,5)P_2_ => PtdIns(3,4,5)P_2_	[23–26]
Inositol-tetrakisphosphate 1-kinase	ITPK1	–	Ins(1,3,4)P_3_ => Ins(1,3,4,5)P_4_ Ins(1,3,4,5)P_4_ => Ins(1,3,4,5,6)P_5_ Ins(3,4,5,6)P_4_ => Ins(1,3,4,5,6)P_5_	[32, 33, 35, 85, 108]
Inositol pentakisphosphate 2-kinase	IPPK	Ipk1	Ins(1,3,4,5,6)P_5_ => InsP_6_	[27, 28]
Inositol hexakisphosphate kinase	IP6K1,2,3	Kcs1	InsP_6_ => 5PP-InsP_5_ 1PP-InsP_5_ => (1,5)PP_2_-InsP_4_ Ins(1,3,4,5,6)P_5_ => PP-InsP_3_	[25, 42, 83]
Diphosphoinositol pentakisphosphate kinase	PPIP5K1,2	Vip1	InsP_6_ => 1PP-InsP_5_ 5PP-InsP_5_ => (1,5)PP_2_-InsP_4_	[44–47, 109]

## Radioactive Labels for Inositol

Inositol polyphosphates consist of four different atomic species: for example, the chemical formula of the fully phosphorylated inositol ring of InsP_6_ is C_6_H_18_O_24_P_6_. Of these elements, oxygen does not have relevant long-lived radioisotopes and therefore is not used for radiolabelling. Conversely, it is possible to generate useful unstable, and thus radioactive, isotopic species for carbon, hydrogen and phosphorus. We will briefly describe the biophysical characteristics of the most commonly used radioisotopes: tritium [^3^H], carbon [^14^C], and phosphorus [^32^P] and [^33^P] (Table 2). All are radioactive due to β decay.

**Table 2:** Isotopic labels available to generate inositol polyphosphate radioactive tracers

	Symbol	β energy	Range in air	Half-life
Tritium, hydrogen-3	[^3^H]	5.7 keV	<1 cm	12.3 years
Carbon-14	[^14^C]	156 keV	24 cm	5730 years
Phosphorus-32	[^32^P]	1709 keV	610 cm	13.5 days
Phosphorus-33	[^33^P]	249 keV	89 cm	25.4 days

Tritium contains two neutrons as well as the usual hydrogen proton. It is a weak β-emitter with a half-life of over 12 years. The emitted electron has very low energy, and can therefore travel only a few millimetres in air, and cannot penetrate the skin. This makes it particularly easy to work with, as special protective equipment is not required: gloves, goggles and lab coat are sufficient. On the minus side, the emitted radiation is too low to be detected by Geiger counter. Testing for tritium contamination before and after an experiment must instead be performed by swipe testing and liquid scintillation counting. Carbon-14 has a very long half-life, enabling its use in carbon dating in archaeology. The emitted electrons are still reasonably low-energy, although they can be detected using a Geiger counter, and can travel less than 0.3 mm into the skin. Therefore, shielding is also not necessary when working with [^14^C].

Phosphorus-32 is the most energetic radioisotope commonly used in biochemical laboratories. The electrons released are very high-energy and can travel over 6 m through air and 0.76 cm into human tissues. Consequently, extra precautions are required when working with [^32^P]: a 1 cm Plexiglas shield is required for the working area, and dosimeter monitoring for the experimenter. Contamination is easily detected with a Geiger counter. Experiments requiring [^32^P] can also be performed using the superior phosphorus-33 isotope. The emitted radiation from [^33^P] is of lower energy and is therefore less dangerous, although the same safety measures are required. The half-life of [^33^P] is also longer than that of [^32^P]: 25.4 days compared to 14.3 days. This can be extremely beneficial in maximising the value of any radiolabelled compounds synthesised. Only very rare experimental circumstances require the high energy of [^32^P] radiation, but this isotope is still widely used, since there is a huge price difference between the two radioactive isotopes. Phosphorus-33 is much more expensive.

## Inositol Polyphosphate in Vivo Studies Using Radioactive Metabolic Labelling

As mentioned above, the main problem for inositol polyphosphate research is that there is no easy way to detect the inositol ring using spectrophotometry. A technique was developed in the 1980s that combined chromatography, post-column derivatisation and spectrophotometry to visualise the phosphate groups, and thus indirectly the inositol polyphosphate, to try to solve this problem [50]. This method is not sensitive enough to be routinely used with mammalian cells, although there are a few reports of its use [46]; it may be more appropriate for organisms with high levels of inositol polyphosphates such as the amoeba *Dictyostelium discoideum* [3, 51]. In general, for effective, sensitive and reliable methods for detecting and studying the metabolism and many functions of inositol polyphosphates, we must turn to radioactivity.

The standard procedures for investigating inositol polyphosphate metabolism in vivo require the use of radiolabelled inositol tracers [52, 53]. These are usually based on tritium labels, with carbon-14 rarely used, as it is more expensive (Fig. 2b, c). These [^3^H]inositol or [^14^C]inositol tracers (Fig. 2b, c) are added to the extracellular growth medium, where they are taken up by cells and enter the inositol cycle (Figs. 1, 3). Different inositol polyphosphates and phosphoinositides then begin to be radiolabelled. The labelling must be given sufficient time to reach isotopic equilibrium, where all the inositol polyphosphates species are in equilibrium with [^3^H]inositol or [^14^C]inositol. Given the presence of more than 30 inositol polyphosphate species in eukaryote cells [15, 17], and that, for example, seven sequential enzymatic reactions are required to generate InsP_7_, it is clear that allowing time to reach isotopic equilibrium is fundamental to generating reliable experimental data. For yeast, it is normally sufficient to label the cells overnight, which corresponds to 7–8 cell divisions. Unsurprisingly, mammalian cells must be labelled for much longer to reach equilibrium: 4 or 5 days may be enough, depending on cell type. Before starting labelling experiments in a new cell type, a pilot study must be performed to determine the time needed to reach metabolic equilibrium [36]. This is calculated by dividing the radioactivity accumulated in InsP_6_ by the radioactivity of the lipid phosphoinositide pool. This InsP_6_/phosphoinositide ratio increases over time; when it remains constant, the labelling has reached equilibrium. In the modern literature, too often the isotopic equilibrium is not properly calculated or even considered, casting doubt on the reliability of the data generated.

Once metabolic equilibrium has been reached, radiolabelled inositol polyphosphates can be acid-extracted and resolved by strong anion exchange high-performance liquid chromatography (saxHPLC). Two options exist for analysing the samples. Firstly, an in-line radioactivity detector can be used, greatly speeding up analysis and reducing handling at the cost of sensitivity. The alternative is to collect fractions for manual counting with a scintillation β-counter; while more labour-intensive, this method significantly increases the sensitivity. The inositol pyrophosphate species InsP_7_ and InsP_8_ can be easily detected in labelled yeast extracts using manual counting [52, 54], while only the more abundant precursor InsP_6_ has been identified using in-line detectors [28].

It should also be mentioned that dual isotopic labelling is possible. The difference in the energy of electrons emitted by [^3^H]inositol and [^14^C]inositol enables scintillation counters to distinguish these two inositol species. By labelling cells to isotopic equilibrium using [^14^C]inositol, and then briefly with [^3^H]inositol, it is possible to study the possible heterogeneity within pools of phosphoinositides or inositol polyphosphates [55]. This approach was employed successfully in the study of inositol polyphosphates generated after vasopressin or prostaglandin stimulation of vascular tissue. Their source was found to be rapidly labelled phosphoinositides, while the bulk of the highly phosphorylated inositol polyphosphates InsP_5_ and InsP_6_ were not created from the rapid phosphoinositide turnover, and were therefore deemed metabolically inert [56]. Dual labels can also have more technical uses. Chiefly, a second isotopic label is widely used to determine chromatographic saxHPLC peak identity: spiking a [^3^H]inositol labelled extract with a [^14^C]inositol polyphosphate standard enables conclusive identification of the nature of the eluted peaks [24].

The use of commercially available radiolabelled standards would of course be ideal. Unfortunately, many inositol polyphosphates, including InsP_7_, are not available commercially in their radiolabelled form or often even in unlabelled format. The next best option for peak identification is to use the well-characterised yeast inositol kinase mutant strains. Separation of radiolabelled wild-type yeast extracts by saxHPLC reveals a simple elution profile with one major peak of InsP_6_ and two smaller, more polar and therefore later-eluting peaks of InsP_7_ and InsP_8_. These two peaks are absent in extracts from *kcs1*Δ (IP6K mutant) yeast. The *vip1*Δ mutant instead accumulates InsP_7_ [48, 49]. Other mutants show increased peaks for inositol polyphosphates: Ins(1,4,5)P_3_ accumulates in *arg82*Δ multikinase mutants [23], while Ins(1,3,4,5,6)P_5_ accumulates and is converted by Kcs1 into PP-IP_4_ in an *ipk1*Δ mutant. The *ipk1*Δ*kcs1*Δ double mutant has only the increased InsP_5_ peak [54]. Radiolabelled inositol polyphosphate standards can also be generated in vitro enzymatically, whether using radioactive inositol polyphosphate precursors or cold inositol polyphosphates with [^32^P]γATP (as described below). Laboratories with a serious interest in inositol polyphosphate analysis would do well to create standards with a long half-life: extracting and purifying specific inositol polyphosphates from [^3^H]inositol- or [^14^C]inositol-labelled cells gives defined standards that can be used over several years [24, 57].

While radioactive orthophosphate labelling has been instrumental in the early development [58–60] of the inositol phosphate research field, it must be admitted that [^32^P] or [^33^P] orthophosphate labelling currently has limited use for in vivo analysis of inositol polyphosphate metabolism. Many phosphorylated molecules in eukaryotic cells, primarily the abundant nucleotides, are co-purified during the acidic extraction normally employed to purify inositol polyphosphates. Therefore, any chromatogram is essentially undecipherable until the nucleotides have eluted off. The interfering nucleotides can be removed first by charcoal treatment, but this complicates the extraction procedure [61]. For this reason, only a handful of papers published after the 1990s have reported the use of phosphate labelling to study inositol polyphosphate metabolism in vivo. One such use was the original identification of inositol pyrophosphates in *D. discoideum* extracts [3]: the saxHPLC elution region for these high-polarity molecules in this organism is conveniently free from interference from contaminating phosphorylated molecules. This is not the case for extracts from cell types rich in the linear polymer of phosphates [62, 63] inorganic polyphosphate (polyP), such as yeast or trypanosomes, where polyP is particularly abundant [64–66]. In these organisms, orthophosphate labelling experiments will reveal the continuous presence throughout the chromatogram of radiolabelled polyP peaks that cover the InsP_6_, InsP_7_ and InsP_8_ signals (A. Saiardi, unpublished observation).

Orthophosphate labelling using [^32^P] or [^33^P] is more useful in studies of PtdIns lipids. While several types of potentially contaminating phospholipids exist, their number is not as great as the water-soluble (i.e., acid-extracted) phosphorylated molecules present in eukaryotic cytosol. Furthermore, nucleotides are eliminated by the organic solvent extraction procedure required to purify phosphoinositides. Thus it is possible to identify and study radiolabelled phosphoinositides directly, using thin-layer chromatography (TLC) or by resolution by saxHPLC after deacylation [67]. The highly energetic phosphate isotopes also allow the use of in-line radioactive detection methods to measure the saxHPLC-eluted deacylated lipids. It is important to note that orthophosphate [^32^P] or [^33^P] labelling is usually performed over a short period of time, from a few minutes to a few hours only. The phosphorus isotopes are toxic to the cell, and incubation over a few days induces cell stress or even death. Furthermore, orthophosphate labelling does not require reaching any isotopic equilibrium. Tritium or [^14^C] labels are components of the inositol ring itself (Fig. 1b, c), while phosphate labels are not: they are added and removed by the dynamic action of specific phosphatases and kinases, and thus isotopic equilibrium labelling is not necessary.

## Biochemical Synthesis of ^32/33^Phosphate Radiolabelled Inositol Polyphosphates

Even before the cloning of inositol polyphosphate kinases, allowing the synthesis and purification of recombinant enzymes from bacteria, partially purified enzymatic activities or simple cell extracts were used to synthesise radiolabelled inositol polyphosphates [68, 69]. The synthesis of these molecules, labelled with radioactive phosphate at specific positions of the ring, has had numerous applications, including as standards, for monitoring enzymatic activity, or even the discovery of the new post-translational modification protein pyrophosphorylation. Most of the metabolic pathways and functions of lower phosphorylated inositol polyphosphates were elucidated before the cloning of the kinases responsible for their synthesis, often using elegant [^32^P] radiolabelled biochemical assays [70–73]. Studies of the functions of the higher phosphorylated inositol polyphosphates, such as InsP_5_, InsP_6_, and their inositol pyrophosphate derivatives have benefitted from this previous knowledge and by the cloning of their kinases. Using one of the six now known inositol polyphosphate kinases (Table 1) and [^32/33^P]γATP, it is possible to label almost any position of the inositol ring with radioactive phosphate. We will start by discussing the use of recombinant ITPKA, an IP_3_-3 kinase, to generate InsP_4_ labelled at position 3.

## Synthesis of [^32^P]_i_ Radiolabelled InsP_4_ and Its Use

The biosynthesis of [^32^P]InsP_4_ can be achieved by incubating ITPKA with Ins(1,4,5)P_3_ and [^32^P]γATP. This enzyme specifically phosphorylates position 3 of the ring, and thus generates 3[^32^P]Ins(1,3,4,5)P_4_ [74]. Several studies have used this method to investigate the metabolism of this isomer. Notably, its degradation by inositol polyphosphate 5-phosphatases (namely SHIP1/2) can be studied by following the formation of the radiolabelled and thus easily traceable 3[^32^P]Ins(1,3,4)P_5_ [75]. The anabolism of 3[^32^P]Ins(1,3,4,5)P_4_ and conversion to [^32^P]InsP_6_ has also been studied after incubation with nuclear extracts from *D. discoideum* [76].

SHIP2 is more famous for its ability to convert PtdIns(3,4,5)P_3_ to PtdIns(3,4)P_2_, thus regulating the signal from these two important lipids [77]. To study SHIP activity against lipids, radiolabelled [^32^P]PtdInsP_3_ substrate may be required. Previously, PI3Ks were employed to create this, but these are large proteins for which recombinant expression from bacteria is difficult. A current alternative is to use IPMK, which can act on not only the soluble InsP(1,4,5)P_3_ but also the lipid PtdIns(4,5)P_2_ [78], allowing straightforward synthesis of 3[^32^P]PtdIns(3,4,5)P_3_, specifically labelled in position 3 [79]. Human IPMK is easily produced from *Escherichia coli* [26, 78].

## Preparing [^32^P]_i_ Radiolabelled InsP_5_ and Its Use

**Fig. 4:**
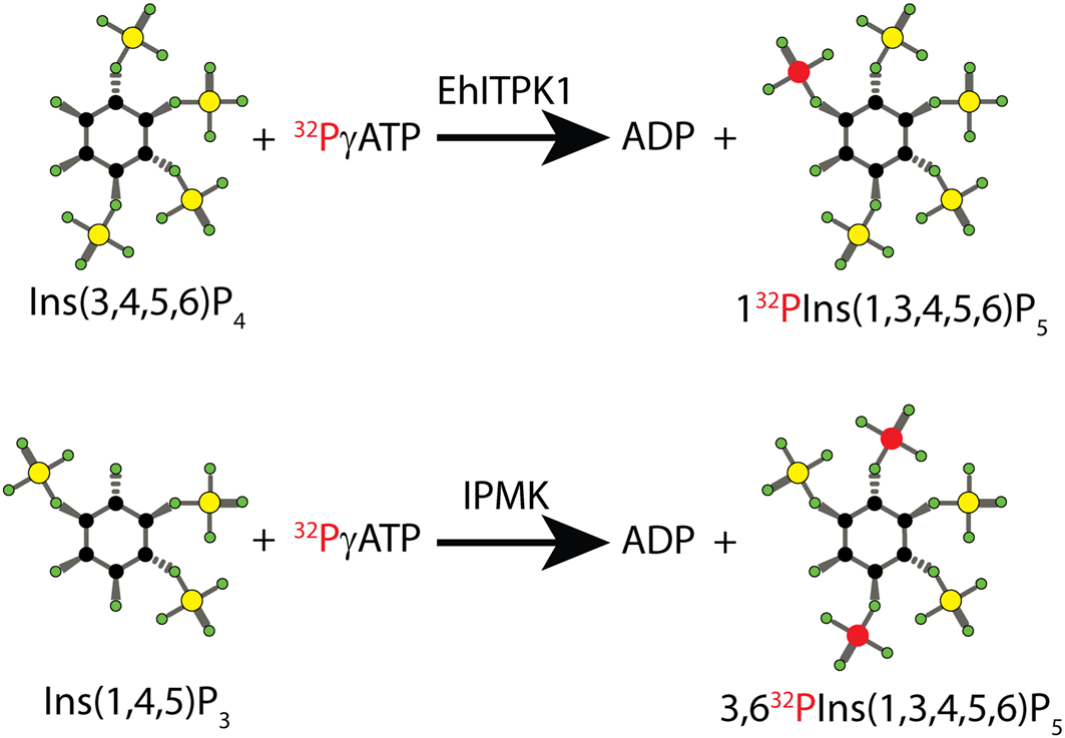
Schematic synthesis of radiolabelled InsP_5_. The most abundant inositol pentakisphosphate isomer, Ins(1,3,4,5,6)P_5_, is the common end product of two multikinases, IPMK and ITPK1, using different starting inositol triphosphates (see Fig. 3). Therefore, using these two enzymes and different species of InsP_3_ and InsP_4_, it is possible to generate InsP_5_ labelled in different positions of the inositol ring. The *top* reaction illustrates the ability of *Entamoeba histolytica* ITPK1 (EhITPK1) to phosphorylate position 1 [34, 35], enabling specific synthesis of 1[^32^P]Ins(1,3,4,5,6)P_5_. Conversely, the *bottom* reaction uses the mammalian IPMK, a 3,6 kinase. Recombinant IPMK can thus be used to generate radiolabelled 3,6[^32^P]Ins(1,3,4,5,6)P_5_ [88]. Different atoms are colour-coded as follows: carbon *black circle*; oxygen *green circle*; phosphate *yellow circle*; radioactive phosphate *red circle*

Several inositol phosphate kinases are quite promiscuous. IPMK, as the name states, is a multikinase able to phosphorylate the inositol ring at positions 4 and 6, but also shows, at least in vitro, the ability to convert InsP_5_ to the inositol pyrophosphate PP-InsP_4_ [26, 80]. As noted above, it is also able to phosphorylate the lipid PtdIns(4,5)P_2_ to PtdIns(3,4,5)P_3_ [78, 81]. The IP6Ks can metabolise several isomers of InsP_5_ and InsP_6_ to inositol pyrophosphates [82, 83]. But perhaps the inositol phosphate kinase most catalytically flexible is ITPK1, which while primarily characterised as a 5- and 6-kinase (Fig. 3) [33], in certain species also has the ability to phosphorylate position 1 of the inositol ring [35]. This activity enables the synthesis of 1[^32^P]Ins(1,3,4,5,6)P_5_ [84] by incubating Ins(3,4,5,6)P_4_ with [^32^P]γATP in the presence of *Entamoeba histolytica* ITPK1 produced in *E. coli* [85] (Fig. 4). Using 1[^32^P]Ins(1,3,4,5,6)P_5_, an intriguing intersubstrate phosphate transfer activity was also discovered for ITPK1. In the presence of 1[^32^P]Ins(1,3,4,5,6)P_5_ and ADP, human ITPK1 transfers the radioactive phosphate, generating [^32^P]γATP and Ins(3,4,5,6)P_4_. The addition of Ins(1,3,4)P_3_ to this reaction augmented the rate of dephosphorylation of 1[^32^P]Ins(1,3,4,5,6)P_5_, as Ins(1,3,4)P_3_ now became the acceptor of the radioactive phosphate group, forming radiolabelled [^32^P]Ins(1,3,4,5/6)P_4_ phosphorylated at the 5 or 6 position, plus again Ins(3,4,5,6)P_4_ [84]. Thus the use of radioactive 1[^32^P]Ins(1,3,4,5,6)P_5_ enabled elucidation of how human ITPK1 regulates the synthesis of Ins(3,4,5,6)P_4_, a signalling molecule fundamental to controlling chloride channel conductance [31, 32]. It was later demonstrated, again using 1[^32^P]Ins(1,3,4,5,6)P_5_, that ITPK1 from the plant *Solanum tuberosum* possesses similar intersubstrate phosphotransferase activity [86].

A different [^32^P]Ins(1,3,4,5,6)P_5_ radiolabelled isomer was initially purified from metabolically labelled cells [72, 87]. It can now be made using IPMK with Ins(1,4,5)P_3_ and [^32^P]γATP, generating 3,6[^32^P]Ins(1,3,4,5,6)P_5_ (Fig. 4). Similarly, [^3^H]Ins(1,3,4,5,6)P_3_ is generated by incubating recombinant IPMK with the commercially available [^3^H]Ins(1,3,4,5)P_4_ and ATP. Both radiolabelled reagents 3,6[^32^P]Ins(1,3,4,5,6)P_5_ and [^3^H]Ins(1,3,4,5,6)P_5_ have been employed to great effect in studying the metabolic stability of InsP_5_ and its anti-tumour capability [88].

## Preparing [^32^P]_i_ Radiolabelled InsP_6_ and Its Use

The commercial availability of [^3^H]InsP_6_ has been intermittent over the years. This is unfortunate, as it was indispensable, for example, in the cloning of the IP6K enzymes from rat brain homogenate: the conversion of [^3^H]IP_6_ to [^3^H]InsP_7_ was followed using polyethylenimine cellulose thin-layer chromatography (PEI-TLC) [25, 68]. The custom synthesis of [^32^P]InsP_6_ was similarly essential to the identification of the other class of enzymes, PPIP5Ks, able to synthesise inositol pyrophosphates. High specific activity [^32^P]InsP_6_ can be generated enzymatically by incubating Ins(1,3,4,5,6)P_5_ and [^32^P]γATP with recombinant *Arabidopsis thaliana* IPK1 enzyme [89]. After HPLC purification, this enzymatic reaction generates [^32^P]IP_6_ specifically labelled in position 2, i.e., 2[^32^P]Ins(1,2,3,4,5,6)P_6_. The use of this compound spurred the identification, cloning and characterisation of the yeast PPIP5K (Vip1), since its conversion to [^32^P]InsP_7_ by *kcs1*Δ (IP6K deletion) yeast extracts revealed the presence of another inositol pyrophosphate synthase activity [47]. Radiolabelled [^32^P]InsP_6_ has also been used to follow IP6K activity during the yeast cell cycle [90] by simply resolving radioactive [^32^P]IP_6_ and [^32^P]IP_7_ by PEI-TLC. In addition to eliminating the need for a sophisticated HPLC apparatus, this experimental approach is quantitative, since the radioactivity present in the [^32^P]InsP_6_ and [^32^P]InsP_7_ TLC spots can be measured by scraping and counting them in a scintillation counter.

## Preparing [^32^P]_i_ Radiolabelled InsP_7_ and Its Use in Protein Pyrophosphorylation Reactions

**Fig. 5:**
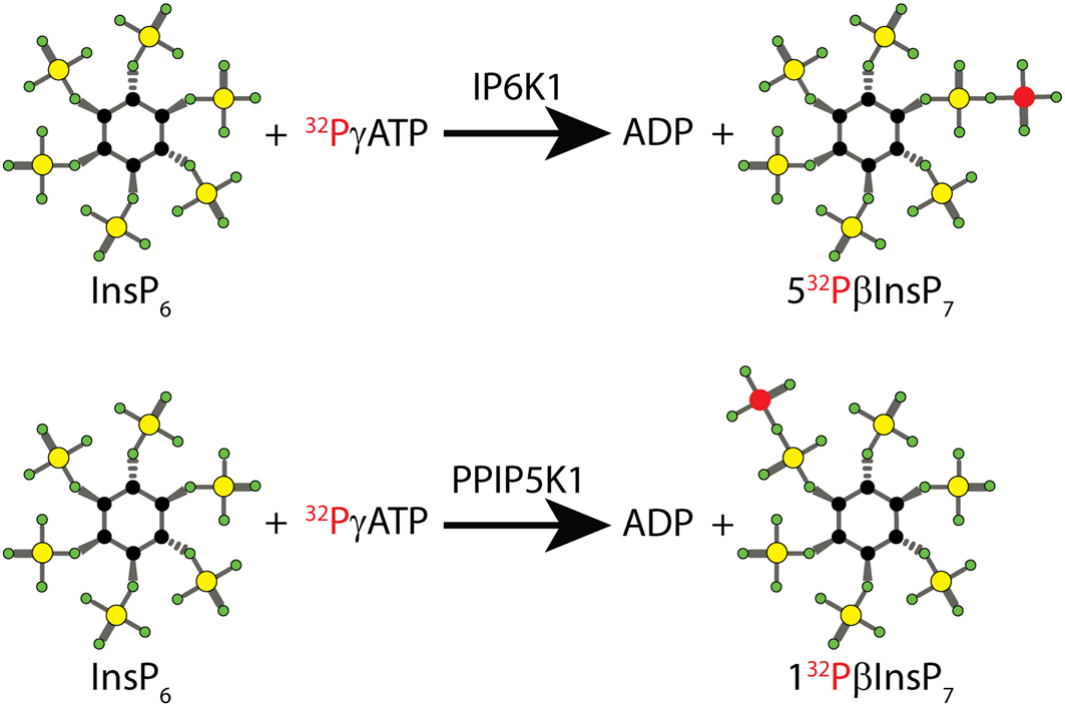
Schematic synthesis of radiolabelled 5[^32^P]βInsP_7_. Two different InsP_7_ isomers can be easily and rapidly synthesised biochemically, using recombinant IP6K1 or the kinase domain of PPIP5K1 (or its yeast counterpart Vip1). Using radiolabelled [^32^P]γATP and InsP_6_ as substrate, IP6K1 generates 5[^32^P]βInsP_7_ (*top*), thus transferring the radioactive [^32^P] from ATP to the phosphorylated position 5 of InsP_6_. PPIP5K instead generates the isomer 1[^32^P]βInsP_7_ (*bottom*). Different atoms are colour-coded as follows: carbon *black circle*; oxygen *green circle*; phosphate *yellow circle*; radioactive phosphate *red circle*

The inositol pyrophosphates InsP_7_ and InsP_8_ have been linked to many cellular roles, but the mechanism is not clear. These are known to be dynamic molecules, unlike their “metabolically inert” precursor InsP_6_ [2]: in mammalian cells, up to 50% of the pool of InsP_6_ may be converted to InsP_7_ or InsP_8_ per hour [91]. One possible mode of action for InsP_7_ is protein pyrophosphorylation, in which the β-phosphate is donated to a pre-phosphorylated serine, becoming InsP_6_ and generating a pyrophosphoserine residue. The discovery of this post-translational modification followed the cloning of IP6K1 [25] and the subsequent ability to synthesise InsP_7_ radiolabelled at the β-position of the pyrophosphate moiety, 5[^32^P]βInsP_7_ (Fig. 5), using InsP_6_ and [^32^P]γATP [92]. Synthesis must be followed by a saxHPLC purification procedure to remove any leftover [^32^P]γATP. Radiolabelled 5[^32^P]βInsP_7_ fractions are then ready to use following desalting with a Sep-Pak QMA cartridge and concentration with a centrifugal evaporator [52]. Experiments demonstrated kinase-independent phosphorylation of multiple proteins in vitro [93]. In the absence of alternative detection methods, the use of the labelled 5[^32^P]β InsP_7_ is still reported in all publications of serine pyrophosphorylation [94–96].

Performing the in vitro pyrophosphorylation/transphosphorylation experiment, once 5[^32^P]βInsP_7_ is synthesised, is a straightforward process. However, it is important to remember the requirement for a pre-phosphorylated serine residue as substrate. Thus proteins of bacterial origin, such as recombinant mammalian proteins expressed in *E. coli*, require a priming event by a classical ATP kinase, usually casein kinase 2 (CK2) [93, 94]. The 5[^32^P]βInsP_7_ pyrophosphorylation is a non-enzymatic, temperature-dependent event that, while it does occur at physiological temperatures, is enhanced by incubating the sample at higher temperatures [97]. Once pyrophosphorylated, proteins can be resolved by polyacrylamide gel electrophoresis (PAGE), and their radioactivity incorporation can be easily identified by autoradiography. Several substrates of InsP_7_-mediated pyrophosphorylation have been identified so far. A common feature to all is stretches of serines embedded in regions rich in acidic residues; as magnesium is required for pyrophosphorylation, it is possible that the acidic residues are required to coordinate these ions. Pyrophosphorylated proteins are more acid-labile but also more resistant to phosphatases than proteins phosphorylated by ATP alone [93]. If this is true in vivo, it could have huge implications for cellular signalling: InsP_7_-mediated protein pyrophosphorylation could act in a dominant manner to allow continued signalling even during phosphatase activation.

It is important to note that inositol pyrophosphates appear to be interchangeable in their ability to pyrophosphorylate proteins. For example, 1[^32^P]βInsP_7_ synthesised using the Vip1 (PPIP5K) kinase domain and [^32^P]γATP (Fig. 5) is able to pyrophosphorylate proteins, as is 1,5[^32^P]InsP_8_ synthesised by the double action of IP6K1 and Vip1. In fact, the incubation of *S. cerevisiae* extracts with 5[^32^P]βInsP_7_, 1[^32^P]βInsP_7_ or 1,5[^32^P]βInsP_8_ revealed an identical pattern of pyrophosphorylated proteins [93]. This indicates that if pyrophosphorylation is the mechanism of action for inositol pyrophosphates, they can substitute for each other in performing their biological role. However, allosteric regulation of proteins has also been proposed as a mechanism [98, 99], and in this circumstance, different isomeric species of inositol pyrophosphates could be recognised by specific effector proteins.

The synthesis described here for 5[^32^P]βInsP_7_ is a straightforward procedure, as is using it to identify pyrophosphorylated proteins. However, processing millicurie amounts of radioactivity to generate a reagent with a two week half-life requires a degree of dedication. Therefore, protein pyrophosphorylation, while considered an exciting post-translational modification, has received little attention, as only a handful of laboratories have invested in the synthesis of 5[^32^P]βInsP_7_. The recent development of organic synthesised experimental tools to study protein pyrophosphorylation [100, 101] will likely lead to the further and very welcome development of non-radioactive detection methods, and to the demonstration of the existence of this modification in vivo. These developments will certainly increase interest in protein pyrophosphorylation, but it must be remembered that it was the innovative use of radioactive labelling methods that permitted the discovery of this modification in the first place. This is an excellent demonstration that original radioactive assays should not be avoided.

## Perspective

In today’s “omics” era, radioactive labelling appears anachronistic. Current proteomic, metabolomic or genomic studies give us plentiful information in an ever-growing number of databases. Ultimately, however, these huge amounts of data must not be just statistically annotated, but must drive biochemistry experiments, which as noted before, provide the only experimental basis for the understanding of biological mechanisms [102, 103]. In this context, radioactive molecular tracers have played and will continue to play a contributory role in our quest to understand the molecular mechanisms of life.

This review has highlighted the fundamental importance of radioactive labelling in the birth and the development of inositol polyphosphate research. Although recent technological development efforts such as PAGE analysis [82] and TiO_2_ extraction of inositol polyphosphates [104] are facilitating the study of the metabolism and functions of these molecules without the need for radioactive precursors or metabolic labelling, we definitely foresee further need for phosphate-32/33-, carbon-14- or tritium-based experiments to fully appreciate the importance of these molecules in cell biology.
